# Lipoprotein(a) and the Risk of Heart Failure: A Dose‐Response Meta‐Analysis

**DOI:** 10.1002/clc.70289

**Published:** 2026-04-07

**Authors:** Yongmei He, Jun Liu, Jingwei Zhuang, Hongjia Hu

**Affiliations:** ^1^ Department of Endocrinology Pengzhou People's Hospital Pengzhou China

**Keywords:** dose‐response, heart failure, incidence, lipoprotein(a), meta‐analysis

## Abstract

**Background:**

Lipoprotein(a) [Lp(a)] is a genetically determined lipoprotein implicated in cardiovascular disease, but its role in heart failure (HF) remains uncertain. Observational studies indicate a link between elevated Lp(a) and HF risk, but the dose‐response relationship remains unexplored. This meta‐analysis aimed to quantify the association between circulating Lp(a) levels and HF incidence.

**Methods:**

A systematic search of PubMed, Embase, and Web of Science identified prospective cohort studies reporting hazard ratios (HRs) for HF incidence across different Lp(a) levels. A random‐effects model was applied to pool effect estimates while accounting for heterogeneity, and restricted cubic splines assessed dose‐response relationships.

**Results:**

Five prospective cohort studies with 400 631 participants were included. During a mean follow‐up duration of 11.0 years, 10 598 (2.6%) patients developed HF. A high Lp(a) level was associated with an increased HF risk (HR: 1.34, 95% CI: 1.14–1.59, *p* < 0.001), with moderate heterogeneity (I² = 69%). Subgroup analysis showed a stronger association in studies using an Lp(a) cutoff of ≥ 50 mg/dL (HR: 1.68) compared to those with a cutoff of < 50 mg/dL (HR: 1.16, p for subgroup difference < 0.01), which completely explained the heterogeneity. The dose‐response analysis revealed a nonlinear association (p for non‐linearity = 0.001). HF risk increased nearly linearly below 55 mg/dL, then slowed, and plateaued at 160 mg/dL.

**Conclusions:**

Elevated Lp(a) is associated with an increased HF risk in a nonlinear pattern, with risk escalation slowing at higher concentrations.

## Graphical abstract

This meta‐analysis found that higher circulating lipoprotein(a) levels are significantly associated with an increased risk of heart failure. The relationship follows a nonlinear pattern, with risk rising at lower concentrations and plateauing at higher levels. These findings highlight lipoprotein(a) as a potential biomarker for heart failure risk stratification.

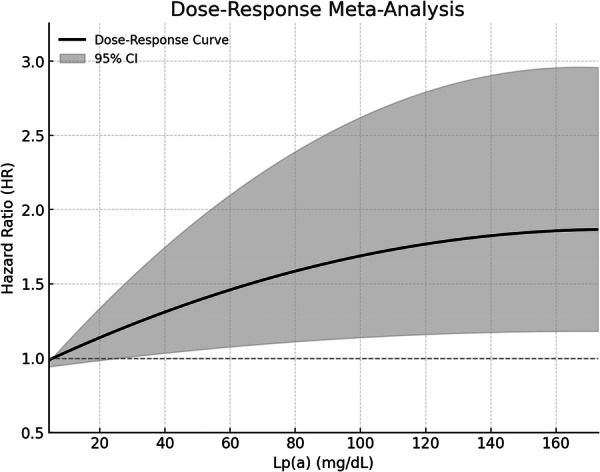

## 1 Introduction

Heart failure (HF) is a leading cause of morbidity and mortality worldwide, affecting over 64 million people globally and placing a significant burden on healthcare systems [1, 2]. Despite advances in pharmacological and device‐based therapies, HF remains a progressive and debilitating condition, with high hospitalization and mortality rates [3]. The 5‐year survival rate after an HF diagnosis is approximately 50%, underscoring the urgent need for improved prevention and early intervention [4]. While established risk factors such as hypertension, diabetes, and coronary artery disease (CAD) play a crucial role in HF development [5], growing evidence suggests that lipid‐related biomarkers, particularly lipoprotein(a) [Lp(a)], may also contribute to HF risk [6, 7]. Identifying novel risk factors is essential for refining HF risk prediction and potentially guiding new therapeutic approaches.

Lp(a) is a genetically determined low‐density lipoprotein (LDL)‐like particle composed of an apolipoprotein(a) [apo(a)] covalently bound to apolipoprotein B‐100 [8, 9]. Unlike traditional lipid markers, Lp(a) levels are primarily inherited, with minimal influence from diet or lifestyle, and remain relatively stable throughout life [8]. Elevated Lp(a) is an established risk factor for atherosclerotic cardiovascular diseases (ASCVD), including myocardial infarction (MI) [10] and stroke [11], and has been implicated in valvular heart diseases such as aortic stenosis [12]. The potential mechanisms linking Lp(a) to HF include its pro‐atherogenic, pro‐inflammatory, and pro‐thrombotic properties, which contribute to microvascular dysfunction, myocardial fibrosis, and left ventricular remodeling, all of which are critical in HF pathogenesis [6, 13]. However, despite the increasing recognition of Lp(a) as a cardiovascular risk marker, its association with incident HF remains unclear, with conflicting results from observational studies [6, 14].

Several observational studies have evaluated the relationship between Lp(a) and HF, yet findings have been variable [15, 16, 17, 18, 19]. Furthermore, most studies dichotomized Lp(a) levels rather than assessing a potential dose‐response relationship, which limits the ability to determine whether a threshold effect exists. As Lp(a) gains recognition in cardiovascular medicine, a dose‐response meta‐analysis is necessary to quantify its association with HF and assess potential nonlinearity. Accordingly, in this study, we performed a meta‐analysis of prospective cohort studies aiming to systematically evaluate the association between circulating Lp(a) levels and the incidence of HF, incorporating a dose‐response analysis to determine whether the relationship follows a linear or nonlinear pattern.

## 2 Methods

This meta‐analysis followed the Preferred Reporting Items for Systematic Reviews and Meta‐Analyses (PRISMA) 2020 guidelines [20, 21] and the Cochrane Handbook for Systematic Reviews and Meta‐Analyses [20] for protocol design, data extraction, statistical analysis, and results reporting. The study protocol was also registered in PROSPERO under ID CRD42025637118.

### 2.1 Literature Search

Relevant studies for this meta‐analysis were identified through a comprehensive search in PubMed, Embase, and Web of Science using a broad range of search terms, which included: (1) “lipoprotein(a)” OR “Lp(a)” OR “Lp[a];” combined with (2) “heart failure” OR “cardiac failure” OR “cardiac dysfunction” OR “ventricular dysfunction” OR “cardiac insufficiency.” Only peer‐reviewed, full‐length articles in English on human studies were included. Additionally, references from relevant original and review articles were manually screened for further eligible studies. The search spanned from database inception to April 23, 2025. The detailed search strategy for each database is shown in File S1.

### 2.2 Inclusion and Exclusion Criteria

The eligibility criteria for studies were established based on the Population, Intervention, Comparison, Outcome, and Study design (PICOS) [22] framework:

P (patients): Adult participants (aged 18 years and older) from general population, community population, or at‐risk populations for HF.

I (exposure): Circulating Lp(a) was measured at baseline, and participants with a high Lp(a) level were considered as exposure, with the cutoff values for defining a high Lp(a) consistent with the original studies.

C (comparison): Participants with a low Lp(a) level at baseline.

O (outcome): Hazard ratios (HRs) and corresponding 95% confidence intervals (CIs) for the incidence of HF during follow‐up, compared between participants with a high versus a low Lp(a) level at baseline. The minimal follow‐up duration was 1 year.

S (study design): Prospective cohort studies.

Studies were excluded if they were reviews, editorials, meta‐analyses, preclinical research, cross‐sectional studies, retrospective studies, studies including pediatric patients, lacked Lp(a) as the exposure, or did not report HF incidence as the outcome of interest. Studies focusing solely on patients with cardiovascular conditions, such as post‐MI patients at high HF risk, were excluded. When population overlap occurred, the study with the largest sample size was selected for inclusion in the meta‐analysis.

### 2.3 Study Quality Assessment and Data Extraction

Two authors independently conducted the literature search, study selection, quality assessment, and data extraction, resolving discrepancies through discussion with the corresponding author. Study quality was evaluated using the Newcastle–Ottawa Scale (NOS) [23], which assesses selection, confounding control, and outcome measurement, with scores ranging from 1 to 9, where 9 represents the highest quality. Studies with NOS scores of 7 or above are considered of high quality. Data extracted for analysis included study characteristics (author, year, country, and design), participants characteristics (source of the population, sample size, mean age, and sex), methods for measuring Lp(a), determining the cutoffs of Lp(a), and cutoff values for defining a high Lp(a) level, mean follow‐up durations, definition of HF outcomes, and numbers of patients who developed HF during follow‐up, and the variables adjusted in the multivariate analysis models when the association between Lp(a) and HF was evaluated.

### 2.4 Statistical Analyses

HRs and 95% CIs were used to summarize the association between Lp(a) levels at baseline and the risk of HF, compared between participants with a high versus a low circulating Lp(a) levels at baseline. HRs and their standard errors were calculated from 95% CIs or p‐values and log‐transformed to stabilize variance and normalize distribution [20]. To assess heterogeneity, we used the Cochrane Q test and I² statistics [24], with I² < 25%, 25% ~ 75%, and > 70% indicating mild, moderate, and substantial heterogeneity among the included studies. A random‐effects model was used to synthesize results while accounting for variability across studies [20]. Sensitivity analysis was conducted by sequentially excluding individual studies to assess the robustness of the findings. Subgroup analyses were performed to evaluate the influences of study characteristics on the results of the meta‐analysis, such as the mean ages of the patients, proportions of men, cutoff values for defining a high Lp(a) level, and follow‐up durations. Subgroups were defined using the median values of continuous variables as cutoff points. In addition, we performed a dose‐response meta‐analysis to derive pooled estimates based on the natural logarithm of HRs and corresponding 95% CIs across different categories of circulating Lp(a) levels. Studies were included if they assessed HF risk across at least three Lp(a) categories. For each category of Lp(a) level, we used the midpoint of Lp(a) levels if the mean or median level per category was not reported. For open‐ended extreme categories, we determined the cut‐off value using the width of the adjacent interval. Linear dose‐response relationships were analyzed following the method of Greenland and Longnecker [25], while nonlinear associations were evaluated using restricted cubic splines with knots at the 10th, 50th, and 90th percentiles [26]. Publication bias was assessed through funnel plots, visual asymmetry inspection, and Egger's regression test [27]. A *p* value < 0.05 indicates statistical significance. The statistical analyses were conducted using RevMan (Version 5.1; Cochrane Collaboration, Oxford, UK) and Stata software (version 12.0; Stata Corporation, College Station, TX, USA).

## 3 Results

### 3.1 Study Identification

**FIGURE 1:**
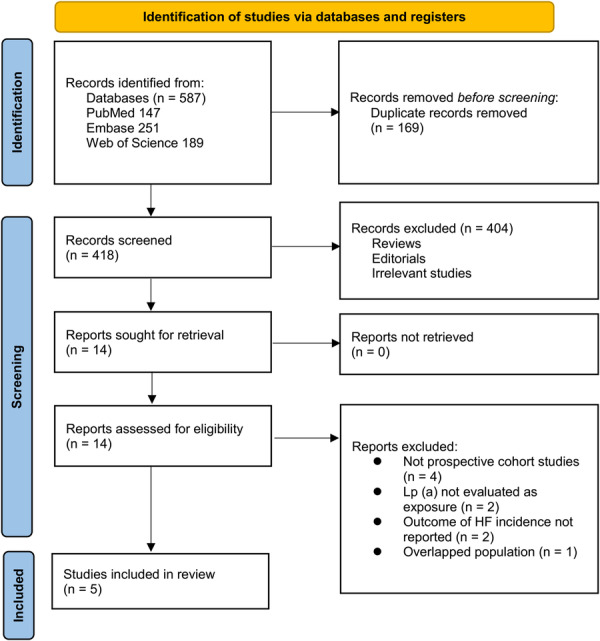
Flowchart of database search and study inclusion. Studies with overlapping or potentially overlapping populations were excluded by retaining only the report with the largest sample size and/or longest follow‐up.

Figure 1 outlines the study selection process. Initially, 587 records were identified across three databases, with 169 duplicates removed. After title and abstract screening, 404 articles were excluded for not meeting the meta‐analysis criteria. The full texts of the remaining 14 studies were independently reviewed by two authors. Nine studies were excluded for reasons specified in Figure 1. For studies derived from the same or potentially overlapping cohorts, only the study with the largest sample size and/or longest follow‐up was retained to avoid double‐counting participants, resulting in five studies included in the final quantitative synthesis. Ultimately, five studies were included in the quantitative analysis [15, 16, 17, 18, 19].

### 3.2 Overview of the Study Characteristics

Table 1 presents a summary of the characteristics of the studies included in the meta‐analysis. Overall, five prospective cohort studies [15, 16, 17, 18, 19] involving 400 631 adults were included in the meta‐analysis. These studies were published from 2016 to 2024, and were conducted in Denmark, the United States, and the United Kingdom. Four studies included participants from community populations [15, 16, 17, 18], and the other one included adults referred for coronary or peripheral angiography [19]. The mean ages of the patients were 55.0–65.0 years, and the proportions of men were 42.7%–70.9%. Circulating Lp(a) was measured with immunoturbidimetric assay in three studies [15, 17, 18], with enzyme‐linked immunosorbent assay [16] and modified immunoassay [19] in the other two studies. The cutoffs for determination of a high Lp(a) level were defined according to the 90th percentile of Lp(a) in one study [15], the fifth quintile in two studies [16, 18], and the previously defined cutoffs in another two studies [17, 19]. The cutoff values for defining a high Lp(a) ranged from 21.3 to 70.0 mg/dL among these studies. The mean follow‐up durations were 3.7–23.4 years. The HF outcome was validated by International Classification of Diseases codes in three studies [15, 16, 18] and via clinical diagnosis in two studies [17, 19]. Overall, 10 598 (2.6%) patients developed HF during follow‐up. All studies performed multivariate analyses adjusting for age, sex, cardiovascular risk factors, and lipid‐lowering treatments, though adjustment levels varied. The NOS scores ranged from seven to nine, indicating high methodological and reporting quality (Table S1).

**TABLE 1:** Characteristics of the included studies.

Study	Country	Design	Population characteristics	No. of subjects	Mean age (years)	Men (%)	Methods for measuring Lp(a)	Methods for determining Lp(a) cutoff	Cutoff value of Lp(a) (mg/dL)	Follow‐up durations (years)	Definition of HF outcomes	No. of patients with HF	Variables adjusted
Kamstrup 2016	Denmark	PC	Community population	98097	58.0	45.0	ITA	> 90th percentile versus < 34th percentile	67.0	7.0	ICD codes evidenced diagnosis of HF	2078	Age, sex, BMI, TC, HDL‐C, TG, smoking, alcohol intake, physical activity, DM, and lipids lowering therapy
Agarwala 2017	USA	PC	Community population aged 45‐64 years	14154	55.0	45.0	ELISA	Q5 versus Q1	23.1	23.4	HF hospitalization evidenced by ICD codes	2605	Age, sex, race, SBP, hypertension, DM, smoking, BMI, HR, HDL‐C, prevalent CAD, and LDL‐C
Steffen 2018	USA	PC	Community population aged 45‐84 years	6809	62.1	47.1	ITA	Previously defined cutoffs (≥ 50 mg/dL vs. < 30 mg/dL)	50.0	13.0	Medical records evidenced HF diagnosis	308	Age, sex, center, education, BMI, smoking, HTN, lipid lowering medication use, SBP, DBP, baseline eGFR, HDL‐C, TC, TG, and DM
Wang 2023	UK	PC	Community population aged 40‐69 years	280857	57.0	42.7	ITA	Q5 versus Q1	21.3	11.8	HF hospitalization evidenced by ICD codes	5502	Age, sex, ethnicity, smoking, BMI, SBP, antihypertensive medication use, HR, and DM
Januzzi 2024	USA	PC	Adults referred for coronary or peripheral angiography	714	65.0	70.9	Modified immunoassay	Previously defined cutoffs (≥ 70 mg/dL vs. < 70 mg/dL)	70.0	3.7	Clinically diagnosed systematic HF	105	Age, sex, hypertension, DM, smoking, TC, HDL‐C, TG, hsCRP, CKD, and ASCVD

Abbreviations: ASCVD, atherosclerotic cardiovascular disease; BMI, body mass index; CAD, coronary artery disease; CKD, chronic kidney disease; DBP, diastolic blood pressure; DM, diabetes mellitus; eGFR, estimated glomerular filtration rate; ELISA, enzyme‐linked immunosorbent assay; HF, heart failure; HDL‐C, high‐density lipoprotein cholesterol; HR, heart rate; hsCRP, high‐sensitivity C‐reactive protein; ICD, International Classification of Diseases; ITA, immunoturbidimetric assay; LDL‐C, low‐density lipoprotein cholesterol; Lp(a), lipoprotein(a); PC, prospective cohort; Q1/Q5, first quintile/fifth quintile; SBP, systolic blood pressure; TC, total cholesterol; TG, triglycerides.

### 3.3 Lp(a) Level and the Risk of HF

**FIGURE 2:**
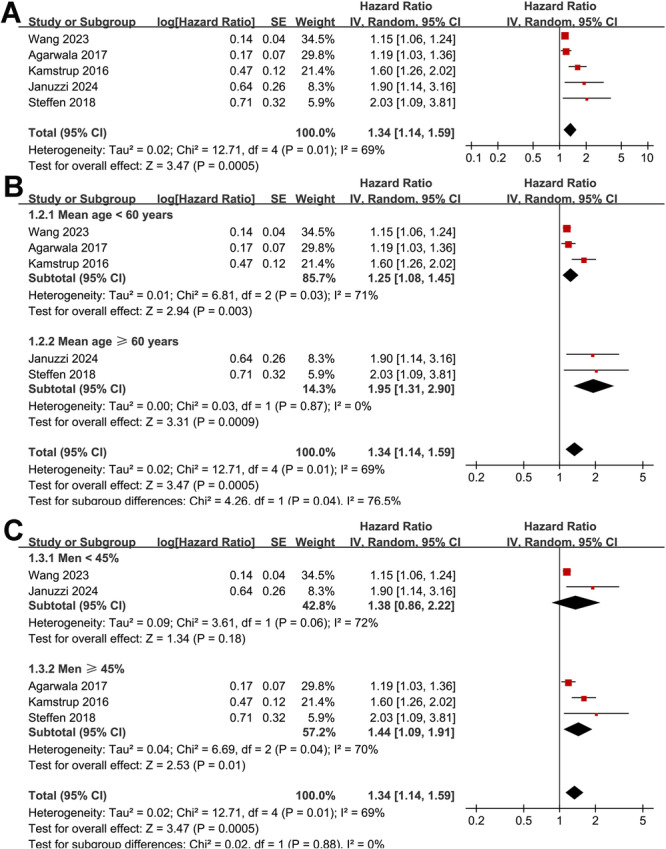
Forest plots for the meta‐analysis of the association between circulating Lp(a) and the risk of HF in adult population; A, overall meta‐analysis; B, subgroup analysis according to the mean age of the participants; and C, subgroup analysis according to the proportion of men of the populations.

**FIGURE 3:**
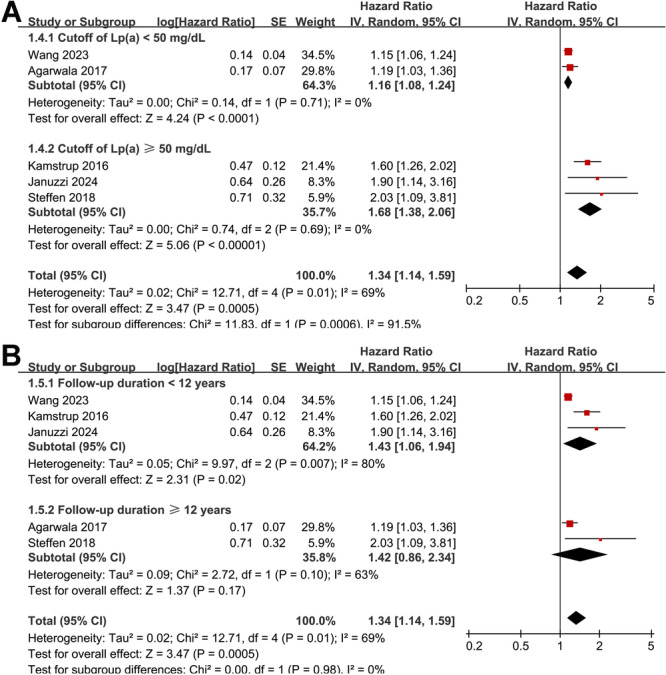
Forest plots for the subgroup analyses of the association between circulating Lp(a) and the risk of HF in adult population; A, subgroup analysis according to the cutoff values for defining a high Lp(a) level in each study; and B, subgroup analysis according to the follow‐up durations.

Overall, the pooled results of five prospective cohort studies using a random‐effects model showed that a high Lp(a) at baseline was associated with an increased risk of HF during follow‐up (HR: 1.34, 95% CI: 1.14–1.59, *p* < 0.001; Figure 2A) with significant and moderate heterogeneity (*p* for Cochrane Q test = 0.01; I^2^ = 69%). Sensitivity analysis, excluding one study at a time, showed no significant impact on the results (HR: 1.24 to 1.51, *p* all < 0.05). Subsequent subgroup analyses suggested that the association between a high Lp(a) level and the increased risk of HF may be more remarkable in participants with mean ages ≥ 60 yeas compared to those < 60 years (HR: 1.95 vs. 1.25, *p* for subgroup difference = 0.04; Figure 2B). The association was not significantly affected by subgroup analysis according to the proportion of men (*p* for subgroup difference = 0.88; Figure 2C). Interestingly, the subgroup analysis results showed a significantly stronger association between Lp(a) and HF in studies with cutoffs for defining a high Lp(a) of ≥ 50 mg/dL as compared to those < 50 mg/dL (HR: 1.68 vs. 1.16, *p* for subgroup difference < 0.01; Figure 3A), which completely explained the source of heterogeneity (I^2^ = 0% for both subgroups). Finally, the subgroup analysis according to the follow‐up durations did not significantly affect the results (*p* for subgroup difference = 0.98; Figure 3B).

### 3.4 Dose‐Response Association Between Lp(a) Level and the Risk of HF

**FIGURE 4:**
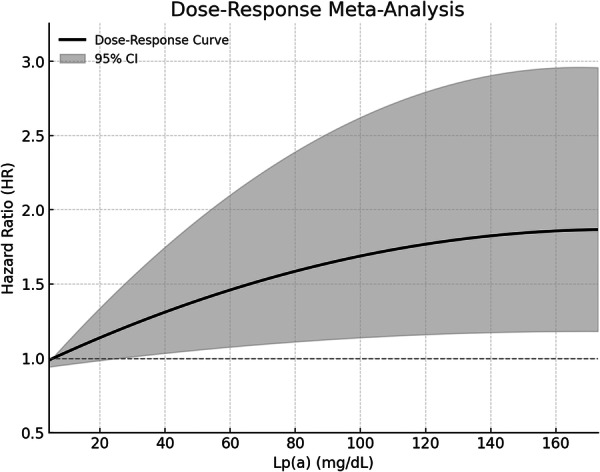
Dose‐response curve for the meta‐analysis of the association between circulating Lp(a) and the risk of HF in adult population.

Four of the included studies reported the association between Lp(a) level and the risk of HF with at least three categories of Lp(a) levels [15, 16, 17, 18], making a total of 14 datasets available for the dose‐response meta‐analysis (detailed in Table S2). The dose–response meta‐analysis revealed an overall nonlinear association between circulating Lp(a) levels and the risk of HF (*p* for nonlinearity = 0.001, Figure 4). At Lp(a) levels below 55 mg/dL, HF risk exhibited a near‐linear increase. At 55 mg/dL, the risk was significantly elevated with an HR of 1.45 (95% CI: 1.05, 2.05). However, beyond 55 mg/dL, the rate of risk elevation gradually slowed, suggesting a diminishing acceleration in HF risk. At 160 mg/dL, the curve entered a plateau phase, with an HR of 1.81 (95% CI: 1.17, 2.80), indicating that further increases in Lp(a) had minimal additional impact on HF risk. This three‐phase pattern shows a linear risk increase at low Lp(a) levels, a gradual slowdown at moderate levels, and a plateau at high concentrations.

### 3.5 Publication Bias

Figure S1 displays funnel plots evaluating the association between Lp(a) levels and the risk of HF. The funnel plots appeared symmetrical on visual inspection, suggesting no obvious small‐study effects. However, formal statistical assessment using Egger's regression test was not performed because only five studies were included, and the power to detect publication bias was therefore limited.

## 4 Discussion

This meta‐analysis of five prospective cohort studies involving 400 631 participants provides comprehensive evidence supporting a significant association between elevated circulating Lp(a) levels and the risk of HF in adult population. The pooled results indicate that individuals with high Lp(a) levels have a 34% increased risk of developing HF. The subgroup analysis identified that the association between Lp(a) and HF risk was more pronounced in studies using a higher Lp(a) cutoff (≥ 50 mg/dL) compared to those using a lower cutoff (< 50 mg/dL), suggesting that the strength of the association may depend on baseline Lp(a) levels. Further, the dose‐response analysis revealed a nonlinear relationship between Lp(a) and HF risk, with a linear increase in risk at Lp(a) levels below 55 mg/dL, followed by a slower rate of risk escalation beyond this threshold and a plateau at approximately 160 mg/dL.

The mechanisms linking Lp(a) to HF risk remain incompletely understood but likely involve multiple interrelated pathophysiological processes. Lp(a) is a highly atherogenic lipoprotein that promotes endothelial dysfunction, systemic inflammation, and thrombogenesis [28, 29], all of which contribute to coronary microvascular dysfunction and myocardial fibrosis, key contributors to HF development [30, 31]. Lp(a) is also enriched in oxidized phospholipids, which trigger pro‐inflammatory and pro‐fibrotic pathways, leading to adverse ventricular remodeling [32]. Furthermore, Lp(a) may directly impair myocardial function by inducing extracellular matrix deposition and collagen accumulation, promoting stiffening of the ventricular wall [33]. These mechanisms provide a biological rationale for the observed association between elevated Lp(a) and increased HF risk, even in individuals without established atherosclerotic cardiovascular disease. The molecular mechanisms underlying the potential association between Lp(a) and the risk of HF deserve further investigation.

The results of the subgroup analyses further highlight the influence of Lp(a) cutoff values and participant characteristics on the observed association. Studies defining high Lp(a) as ≥ 50 mg/dL showed a stronger association with HF risk compared to those using a lower cutoff, suggesting that higher Lp(a) concentrations may have a more pronounced impact on HF development. This finding aligns with prior evidence indicating that Lp(a)‐associated cardiovascular risk is dose‐dependent, with greater elevations conferring higher risk of adverse outcomes [34]. The subgroup analysis by age suggested that the association between Lp(a) and HF risk was stronger in older populations (≥ 60 years) compared to younger individuals, though this difference did not reach statistical significance. This may reflect the cumulative impact of Lp(a)‐mediated vascular and myocardial changes over time, leading to a greater susceptibility to HF in older individuals [35]. However, given the limited number of included studies, the subgroup findings should be interpreted with caution, and further research is warranted to validate these observations.

The dose‐response analysis revealed a nonlinear association between Lp(a) and HF risk, providing important insights into the trajectory of risk elevation. At Lp(a) levels below 55 mg/dL, HF risk increased linearly, suggesting that even modest elevations in Lp(a) may contribute to HF development. However, beyond 55 mg/dL, the rate of risk escalation slowed, and at approximately 160 mg/dL, the curve plateaued, suggesting that further increases in Lp(a) did not translate into proportionally higher HF risk. This pattern may reflect biological saturation of Lp(a)‐related pathogenic pathways, such as endothelial dysfunction, inflammation, and myocardial fibrosis, whereby downstream effects reach a ceiling at very high concentrations [36]. In addition competing cardiovascular risks at extreme Lp(a) levels such as fatal atherosclerotic events occurring before HF onset—may reduce the observable incidence of HF, thereby contributing to the apparent risk plateau in long‐term cohort studies. The clinical implications of these findings warrant further investigation, particularly regarding whether specific Lp(a) thresholds should be considered in HF risk stratification.

This meta‐analysis has several key strengths when interpreted alongside prior Mendelian randomization evidence. While MR studies, such as the meta‐analysis by Singh et al. [14]. strengthen causal inference by leveraging genetic instruments, they rely on assumptions of instrument validity and typically yield modest effect sizes that may not directly translate to clinical risk prediction. In contrast, our study synthesizes prospective cohort data to evaluate observed circulating Lp(a) levels, enabling dose–response modeling and identification of nonlinear risk patterns that are directly relevant to clinical practice. However, unlike MR analyses, residual confounding cannot be fully excluded in observational cohorts, and causality cannot be definitively established. Together, these approaches provide complementary insights, with MR supporting causality and cohort‐based analyses informing risk stratification and potential clinical thresholds. In addition, current meta‐analysis is based on an extensive literature search, incorporating only high‐quality prospective cohort studies, which enhances the validity of the findings by minimizing recall and selection bias [37]. Second, the inclusion of subgroup analyses based on age and Lp(a) cutoffs allows for a more nuanced interpretation of risk stratification, providing clinically relevant insights. Third, the dose‐response meta‐analysis enables a quantitative assessment of risk across the entire Lp(a) spectrum, addressing limitations of prior studies that relied on binary Lp(a) classifications. These strengths collectively support the robustness of the findings. However, several limitations should be acknowledged. However, several limitations should be acknowledged. First, the number of included studies was relatively small, which limited the ability to perform detailed subgroup analyses and reduced the statistical power to formally assess publication bias, despite no obvious asymmetry observed in funnel plots. Second, although the subgroup analysis provided valuable insights, the results should be interpreted with caution, as they may be influenced by differences in study populations, Lp(a) measurement methods, and follow‐up durations. Third, although multiple confounders were adjusted for, residual confounding from unmeasured factors (e.g., genetic variations [38], cardiovascular therapies [39]) remains possible. Fourth, due to the observational nature of the included studies, this meta‐analysis cannot establish causality between Lp(a) and HF risk. Finally, as this analysis was based on study‐level data, rather than individual patient‐level data, more precise risk estimations stratified by sex, race, comorbid conditions, and the types of HF could not be performed. Future individual participant data meta‐analyses may help overcome these limitations.

From a clinical perspective, these findings suggest that elevated Lp(a) may serve as a marker of increased HF risk, particularly in individuals with markedly high concentrations. Current lipid management guidelines already recommend measuring Lp(a) at least once in adulthood to improve atherosclerotic cardiovascular risk stratification; however, Lp(a) testing is not currently incorporated into HF‐specific risk assessment algorithms or guidelines [40]. Given the observational nature of the available evidence and the lack of intervention studies demonstrating that Lp(a)‐lowering reduces HF incidence, routine use of Lp(a) measurement solely for HF risk prediction cannot yet be recommended. Instead, Lp(a) testing may be most informative in selected individuals with high inherited cardiovascular risk, where it could complement established HF risk factors rather than replace them. Despite the development of Lp(a)‐targeting therapies like antisense oligonucleotides and siRNA [41, 42, 43], their effect on HF incidence remains uncertain. Future research should focus on clarifying Lp(a)‐mediated mechanisms in HF, identifying subpopulations that may benefit from targeted interventions, and exploring whether incorporating Lp(a) into HF risk prediction models enhances clinical decision‐making.

## 5 Conclusions

In conclusion, this meta‐analysis provides strong evidence supporting an association between elevated Lp(a) and increased HF risk, with a nonlinear dose‐response relationship characterized by a linear increase in risk at lower Lp(a) levels, a slowing rise at moderate levels, and a plateau at higher concentrations. These findings underscore the need for further research on Lp(a)‘s role in HF pathogenesis and risk stratification, with implications for precision medicine.

## Author Contributions

Yongmei He and Hongjia Hu designed the study. Yongmei He and Jun Liu performed literature search, study identification, and study quality evaluation. Yongmei He and Jingwei Zhuang performed data extraction, statistical analysis, and results interpretation. Yongmei He drafted the manuscript. All authors revised the manuscript and approved the submission.

## Funding

The authors have nothing to report.

## Ethics Statement

The authors have nothing to report.

## Consent

The authors have nothing to report.

## Conflicts of Interest

The authors declare no conflicts of interest.

## Supporting information


**Supporting File S1:** Funnel plots for estimating the potential publication bias underlying the meta‐analysis of the association between circulating Lp(a) and the risk of HF in adult population.

**Supporting Table S1:** Study quality evaluation via the Newcastle‐Ottawa Scale.

**Supporting Table S2:** Data used for dose‐response meta‐analysis.

Supplemental File 1.

## Data Availability

The datasets generated and/or analyzed during the current study are available from the corresponding author on reasonable request.
