# Supplementary material for: Lipoprotein(a) and the Risk of Heart Failure: A Dose‐Response Meta‐Analysis
Source: Clin Cardiol. 2026 Apr 7;49(4):e70289. doi: 10.1002/clc.70289 (PMC13054834; doi:10.1002/clc.70289)

**Supplemental File 1** Funnel plots for estimating the potential publication bias underlying the meta-analysis of the association between circulating Lp(a) and the risk of HF in adult population;


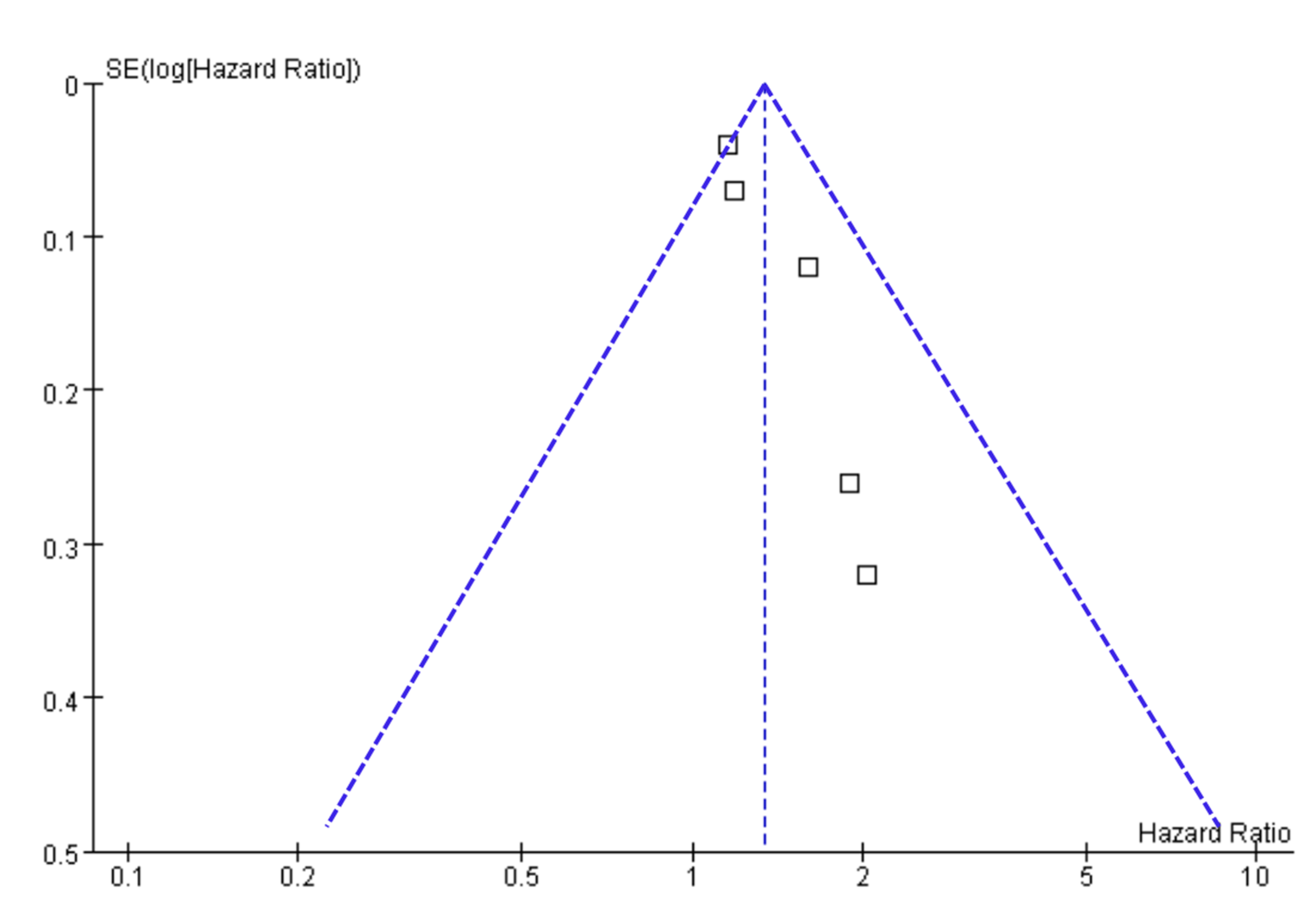

Supplement: Supplementary file 1 — Supporting File S1: Funnel plots for estimating the potential publication bias underlying the meta‐analysis of the association between circulating Lp(a) and the risk of HF in adult population. [file CLC-49-e70289-s001.docx]
