# Supplementary material for: Lipoprotein(a) and the Risk of Heart Failure: A Dose‐Response Meta‐Analysis
Source: Clin Cardiol. 2026 Apr 7;49(4):e70289. doi: 10.1002/clc.70289 (PMC13054834; doi:10.1002/clc.70289)
Supplement: Supplementary file 2 — Supporting Table S1: Study quality evaluation via the Newcastle‐Ottawa Scale. [file CLC-49-e70289-s004.docx]

Supplemental Table 1 Study quality evaluation via the Newcastle-Ottawa Scale

| Cohort study | Representativeness of the exposed cohort | Selection of the non-exposed cohort | Ascertainment of exposure | Outcome not present at baseline | Control for age and sex | Control for other confounding factors | Assessment of outcome | Enough long follow-up duration | Adequacy of follow-up of cohorts | Total |
| --- | --- | --- | --- | --- | --- | --- | --- | --- | --- | --- |
| Kamstrup 2016 | 1 | 1 | 1 | 1 | 1 | 1 | 0 | 1 | 1 | 8 |
| Agarwala 2017 | 1 | 1 | 1 | 1 | 1 | 1 | 0 | 1 | 1 | 8 |
| Steffen 2018 | 1 | 1 | 1 | 1 | 1 | 1 | 1 | 1 | 1 | 9 |
| Wang 2023 | 1 | 1 | 1 | 1 | 1 | 1 | 0 | 1 | 1 | 8 |
| Januzzi 2024 | 0 | 1 | 1 | 1 | 1 | 1 | 1 | 0 | 1 | 7 |
