# Supplementary material for: Lipoprotein(a) and the Risk of Heart Failure: A Dose‐Response Meta‐Analysis
Source: Clin Cardiol. 2026 Apr 7;49(4):e70289. doi: 10.1002/clc.70289 (PMC13054834; doi:10.1002/clc.70289)
Supplement: Supplementary file 3 — Supporting Table S2: Data used for dose‐response meta‐analysis. [file CLC-49-e70289-s002.docx]

Supplemental Table 2 Data used for dose-response meta-analysis

| Datasets | Lp(a) dose (mg/dL) | HR | LL | UL |
| --- | --- | --- | --- | --- |
| Kamstrup 2016-1 | 12 | 1.1 | 0.97 | 1.25 |
| Kamstrup 2016-2 | 38 | 1.24 | 1.08 | 1.42 |
| Kamstrup 2016-3 | 86 | 1.57 | 1.32 | 1.87 |
| Kamstrup 2016-4 | 173 | 1.79 | 1.18 | 2.73 |
| Agarwala 2017-1 | 4.07 | 1.04 | 0.91 | 1.19 |
| Agarwala 2017-2 | 8.49 | 1.1 | 0.96 | 1.25 |
| Agarwala 2017-3 | 17.2 | 1.22 | 1.07 | 1.38 |
| Agarwala 2017-4 | 65.67 | 1.19 | 1.05 | 1.36 |
| Steffen 2018-1 | 40 | 1.53 | 0.87 | 2.7 |
| Steffen 2018-2 | 100 | 2.04 | 1.08 | 3.84 |
| Wang 2023-1 | 4.62 | 0.98 | 0.9 | 1.07 |
| Wang 2023-2 | 8.61 | 1.03 | 0.95 | 1.12 |
| Wang 2023-3 | 20 | 1.06 | 0.97 | 1.15 |
| Wang 2023-4 | 54.7 | 1.15 | 1.06 | 1.25 |

Lp(a), lipoprotein(a); HR, hazard ratio; LL, lower limit of 95% confidence intervals; UL, upper limit of 95% confidence intervals;
