# Supplementary material for: Lipoprotein(a) and the Risk of Heart Failure: A Dose‐Response Meta‐Analysis
Source: Clin Cardiol. 2026 Apr 7;49(4):e70289. doi: 10.1002/clc.70289 (PMC13054834; doi:10.1002/clc.70289)
Supplement: Supplementary file 4 — Supplemental File 1. [file CLC-49-e70289-s003.docx]

**Detailed search strategy for each database**

**PubMed**

("Lipoprotein(a)"[Mesh] OR "lipoprotein(a)" OR "Lp(a)" OR "Lp[a]") AND ("Heart Failure"[Mesh] OR "heart failure" OR "cardiac failure" OR "cardiac dysfunction" OR "ventricular dysfunction" OR "cardiac insufficiency")

**Embase**

('lipoprotein a'/exp OR 'lipoprotein(a)' OR 'Lp(a)' OR 'Lp[a]') AND ('heart failure'/exp OR 'heart failure' OR 'cardiac failure' OR 'cardiac dysfunction' OR 'ventricular dysfunction' OR 'cardiac insufficiency') AND [humans]/lim AND [clinical study]/lim AND [embase]/lim

**Web of Science**

TS=("lipoprotein(a)" OR "Lp(a)" OR "Lp[a]") AND TS=("heart failure" OR "cardiac failure" OR "cardiac dysfunction" OR "ventricular dysfunction" OR "cardiac insufficiency")
